# Dancing cheek to cheek: *Cryptococcus neoformans* and phagocytes

**DOI:** 10.1186/s40064-015-1192-3

**Published:** 2015-08-12

**Authors:** Mingshun Zhang, Donglei Sun, Meiqing Shi

**Affiliations:** Division of Immunology, Virginia-Maryland Regional College of Veterinary Medicine, University of Maryland, College Park, MD USA; Department of Immunology, Nanjing Medical University, Nanjing, Jiangsu China

**Keywords:** *Cryptococcus neoformans*, Macrophage, Neutrophil, Dendritic cell, Endothelial cell

## Abstract

Meningoencephalitis caused by *Cryptococcus neoformans* (Cn) has become one of the leading causes of mortality in AIDS patients. Understanding the interactions between Cn and phagocytes is fundamental in exploring the pathogenicity of cryptococcal meningoencephalitis. Cn may be extracellular or contained in the monocytes, macrophages, neutrophils, dendritic cells and even endothelial cells. The internalized Cn may proliferate inside the host cells, or cause the lysis of host cells, or leave the host cells via non-lytic exocytosis, or even hijack the host cells (Trojan horse) for the brain dissemination, which are regulated by microbe factors and also immune molecules. Coexistence of protective and deleterious roles of phagocytes in the progression of cryptococcosis warrant further investigation.

## Background

Cn has been co-evolved with the phagocyte predators, e.g., amoebas (Chrisman et al. 2010), paramecium (Frager et al. 2010), or nematodes (Casadevall et al. 2003), for a long history. As mammalian phagocytes may originate from the common ancestors, it is plausible to speculate that roles of host phagocytes against Cn manifest the complex interactions between fungi and phagocyte predators (Chrisman et al. 2010). Ideally, predators and their prey fight with each other and maintain the fine balance of nature, implying that neither the prey (Cn) nor the predators (e.g., amoeba, or host phagocytes) would be totally extinguished. Therefore, in the host both Cn and phagocytes would survive, leading to a latent infection, as evidenced by the recent research (Alanio et al. 2015).

Cryptococcal meningoencephalitis occurs only when Cn leaves the infected lung, transmigrates across the blood–brain-barrier (BBB) and proliferates in the brain parenchyma. As Cn is a facultative intracellular pathogen, it is speculated that the transmigrating Cn might be extracellular or within some phagocytes, thereby invading the central nervous system (CNS) via a trans-cellular pathway or Trojan horse pathway (Casadevall 2010). In the trans-cellular pathway, Cn is directly internalized by brain endothelial cells via endocytosis. In the Trojan horse pathway, some phagocytes carrying Cn enter the CNS. High-affinity Fcγ receptor 3A promotes the phagocytosis and significantly contributes to the cryptococcal meningoencephalitis (Rohatgi et al. 2013). Moreover, effective phagocytosis of Cn by macrophages counterintuitively predisposes to poor outcome (Sabiiti et al. 2014), confirming the link between phagocytosis of Cn and the high mortality in patients with cryptococcal meningoencephalitis (Alanio et al. 2011). Together, these data suggest that phagocytes may help Cn invade the CNS. In this review, the interactions between Cn and phagocytes (monocytes, macrophages, neutrophils, dendritic cells, and endothelial cells) are discussed.

## Monocytes and macrophages

**Fig. 1:**
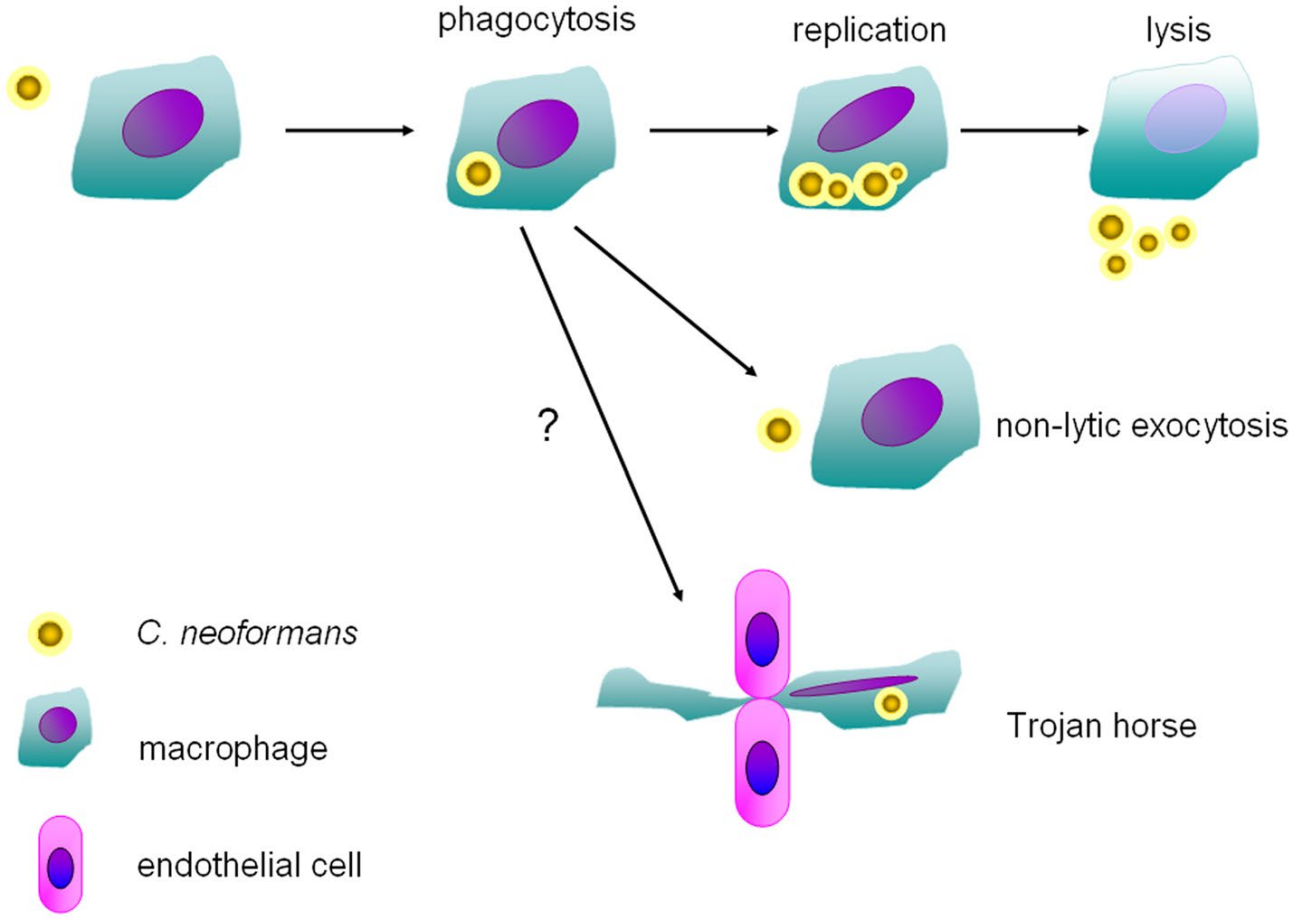
Roles of monocytes in the Cn pathogenesis. Upon infection, monocytes internalize and kill the Cn. However, Cn could also proliferate in the monocytes and escape the monocytes via the host cell lysis or non-lytic exocytosis. It is speculated that monocyte may also work as Trojan horse in the Cn brain dissemination.

Circulating Cn could be detected in monocytes collected from peripheral blood or located in monocytes in the leptomeningeal capillaries. Besides, Cn also could be observed in macrophages in the leptomeningeal space, implying that monocytes and macrophages may play crucial roles in the pathogenesis of cryptococcal meningoencephalitis (Chretien et al. 2002). The outcomes of Cn interacting with macrophages include at least phagocytosis, replication, and non-lytic exocytosis (Coelho et al. 2014; Garcia-Rodas and Zaragoza 2012; Leopold Wager and Wormley 2014; McQuiston and Williamson 2012), implicating the existence of Trojan horse pathway for the brain dissemination (Casadevall 2010; Charlier et al. 2009) (Fig. 1).

### Phagocytosis

Phagocytosis of Cn by macrophages is mediated by diverse factors, including complement proteins, specific antibodies, surfactant protein D (Geunes-Boyer et al. 2009, 2012) or the scavenger receptors SCARF1 and CD36 (Means et al. 2009). IgM and IgA specific to the capsular glucuronoxylomannan (GXM) promote complement-independent and CD18-dependent phagocytosis (Taborda and Casadevall 2002). Phagocytosis of Cn by lung macrophages is significantly impaired in the sIgM deficient mice (Subramaniam et al. 2010). Different from IgG1, IgM and IgA, IgG3-mediated phagocytosis is not associated with FcγR and CD18 (Saylor et al. 2010). In contrast, antiphagocytic protein 1 (App1) from Cn, binding with CR2/CR3, inhibits the phagocytosis of macrophages (Stano et al. 2009; Williams and Del Poeta 2011).

### Replication

As a facultative intracellular pathogen, Cn replicates in and alkalifies the phagosome of macrophages, leading to phagosome breakage and macrophage lysis (Tucker and Casadevall 2002). Replication of Cn inside the macrophages requires F-box protein 1 and its substrate inositol phosphosphingo lipid-phospholipase C1 (Liu and Xue 2014). In addition, Cn phospholipase B1 (PLB1) promotes the survival of fungi in the macrophages by facilitating fungal eicosanoid production (Noverr et al. 2003). In addition, Cn proliferation may stimulate the abortive mitosis (Coelho et al. 2012) of some macrophages (Luo et al. 2005). Mechanisms behind the balance of Cn replication and macrophage lysis/mitosis, however, still remain elusive. Interestingly, a recent study on the dynamics of interactions between Cn and macrophages suggested that fungal background influences outcome during cryptococcal meningoencephalitis in humans (Alanio et al. 2011).

### Non-lytic exocytosis

Besides breaking down the host macrophage, Cn could also escape from macrophages through non-lytic exocytosis or phagosome extrusion in vitro (Alvarez and Casadevall 2006; Ma et al. 2006) or in vivo (Nicola et al. 2011). Virulence factors from fungi, for example, secreted PLB1 and SEC14, are essential for non-lytic exocytosis (Chayakulkeeree et al. 2011). Host factors also regulate the non-lytic exocytosis. The addition of the weak bases ammonium chloride and chloroquine resulted in a significant increase of non-lytic exocytosis events, whereas the vacuolar ATPase inhibitor bafilomycin A1 had the opposite effect (Nicola et al. 2011). Interestingly, both phagosomal maturation dependent (Alvarez and Casadevall 2006) and independent (Ma et al. 2006) pathways have been reported for non-lytic exocytosis of Cn by macrophages. Arp2/3 complex-mediated actin polymerization has been show to inhibit non-lytic exocytosis (Johnston and May 2010). Antibody or complement, which mediates the phagocytosis, affects the outcome (Alvarez et al. 2008) but not the occurrence of non-lytic exocytosis (Alvarez and Casadevall 2006; Ma et al. 2006). Interestingly, autophagy knockdown increases the non-lytic exocytosis of Cn by macrophages (Nicola et al. 2012). Th1 and Th17 cytokines decrease the non-lytic exocytosis, while Th2 cytokines augment the extrusion of Cn out of macrophages (Voelz et al. 2009), which may contribute to the extravasation of Cn and the aggravation of the disease.

### Trojan horse

It has been well documented that monocytes could transmigrate across the BBB and differentiate into perivascular macrophages. Thereby, it is tempting to hypothesize that monocyte harboring Cn might function as Trojan horse in the Cn brain dissemination. Phagocytosis of Cn inhibits the chemotaxis of macrophages stimulated by CX3CL1 and CSF-1 (Luo et al. 2009), which might slow down the crawling of macrophages containing Cn along the brain vasculature and therefore facilitate Cn transmigration. More compelling evidence for Trojan horse comes from the deliberate experiment showing that brain fungal burdens following injection with Cn internalized by macrophages, compared with free Cn inoculation, are significantly higher (Charlier et al. 2009). However, many issues for Trojan horse in Cn brain dissemination, including a direct observation rather the evaluation based on fungus quantification, and the mechanisms behind, still remain unresolved.

## Neutrophils

It has been historically recognized that neutrophils have the ability to kill the Cn, participating in the first-line defenses before a cell-mediated immune response develops (Diamond et al. 1972; Lehrer and Ladra 1977). In vitro, neutrophil kills Cn effectively especially combined with granulocyte colony-stimulating factor (G-CSF) or granulocyte–macrophage colony stimulating factor (GM-CSF) (Chiller et al. 2002). In the murine model of cryptococcosis, G-CSF, if combined with fluconazole, is associated with the increased survival, suggesting that neutrophils contribute to host defenses in cryptococcal meningoencephalitis (Graybill et al. 1997). Administration of G-CSF into the AIDS patients increases the fungicidal activity and decreases the risk of infection (Vecchiarelli et al. 1995), which is associated with the enhanced leukotrienes from neutrophils upon G-CSF therapy (Coffey et al. 1998). In contrast, cryptococcosis is not usually associated with human neutropenia or with conditions characterized by defective neutrophil function (Casadevall and Perfect 1998), reflecting the complexity of roles of neutrophils against Cn. We hypothesize the blurring effects of neutrophils are due to the co-existing protective (positive) and deleterious (negative) roles from neutrophils in the Cn pathogenesis.

Cn or the capsular polysaccharide glucuronoxylomannan (GXM) promotes the inflammatory cytokines (Retini et al. 1996) and chemokines (Lipovsky et al. 1998), thus displaying chemotactic activity on the neutrophils (Dong and Murphy 1993, 1995a). Paradoxically, GXM inhibits neutrophil migration or infiltration (Dong and Murphy 1995b), partially by reducing the L-selectin (Dong and Murphy 1996), E-selectin (Ellerbroek et al. 2002), IL-8 receptor (Lipovsky et al. 1998) of the neutrophils via cross-desensitization, or competitively binding with CD14 (Ellerbroek et al. 2004b), TLR4 (Ellerbroek et al. 2004b), CD18 (Dong and Murphy 1997) on the neutrophils. O-acetylation of GXM is a crucial motive for the inhibition of neutrophil recruitment (Ellerbroek et al. 2004a). Nevertheless, inoculation of Cn intravenously recruits neutrophils accumulated in pulmonary vessels, which is dependent on the complement 5a (C5a) (Lovchik and Lipscomb 1993). Neutrophils recruitment into the lung is also observed at the early phase (Abe et al. 2000; Feldmesser et al. 2000; Herring et al. 2005; Kawakami et al. 1999; Mednick et al. 2003) of Cn airway infection, although it might not be as evident as mononuclear cells in the mice infected with low-virulence strain (Feldmesser et al. 2000; Huffnagle et al. 1998). Recruitment of neutrophils into the lung is dependent on the chemokines including IL-8 (Guillot et al. 2008), MIP-2 and KC (Kawakami et al. 1999), which are elevated upon Cn infection. Cn could also negatively regulate the influx of neutrophils into the lung (O’Meara et al. 2013), deliberately reflecting the paradoxically dual roles in the interaction between neutrophils and Cn.

The recruited neutrophils in the lung internalize Cn after intratracheal inoculation (Feldmesser et al. 2000), which is mediated by complement 3 (C3) (Kozel et al. 1984). Neutrophils activation enhances the phagocytosis (Kozel et al. 1987); while capsule of Cn inhibits the phagocytosis by neutrophils (Richardson et al. 1993). Cn is a facultative intracellular pathogen in the macrophages (Feldmesser et al. 2000). It is largely unknown the definite fate of Cn ingested by neutrophils. Although speculatively Cn might be protected in the neutrophils (Mednick et al. 2003), most research focused on the killing of Cn (Miller and Mitchell 1991), which might happen intracellularly or extracellularly (Qureshi et al. 2010, 2011), in a oxidative-dependent or oxidative-independent manner (Qu and Wang 1991). Myeloperoxidase (MPO) is a neutrophil-specific enzyme closely associated with reactive-oxygen species. MPO-deficient mice infected with Cn intranasally or intravenously survive significantly shorter, due to the impaired clearance of fungus in the lung and the spleen (Aratani et al. 2006). Inhibition of sphingomyelin synthase (SMS) also profoundly impairs the ability of neutrophils to kill Cn, which are independent of phagocytosis (Qureshi et al. 2010).

**Fig. 2:**
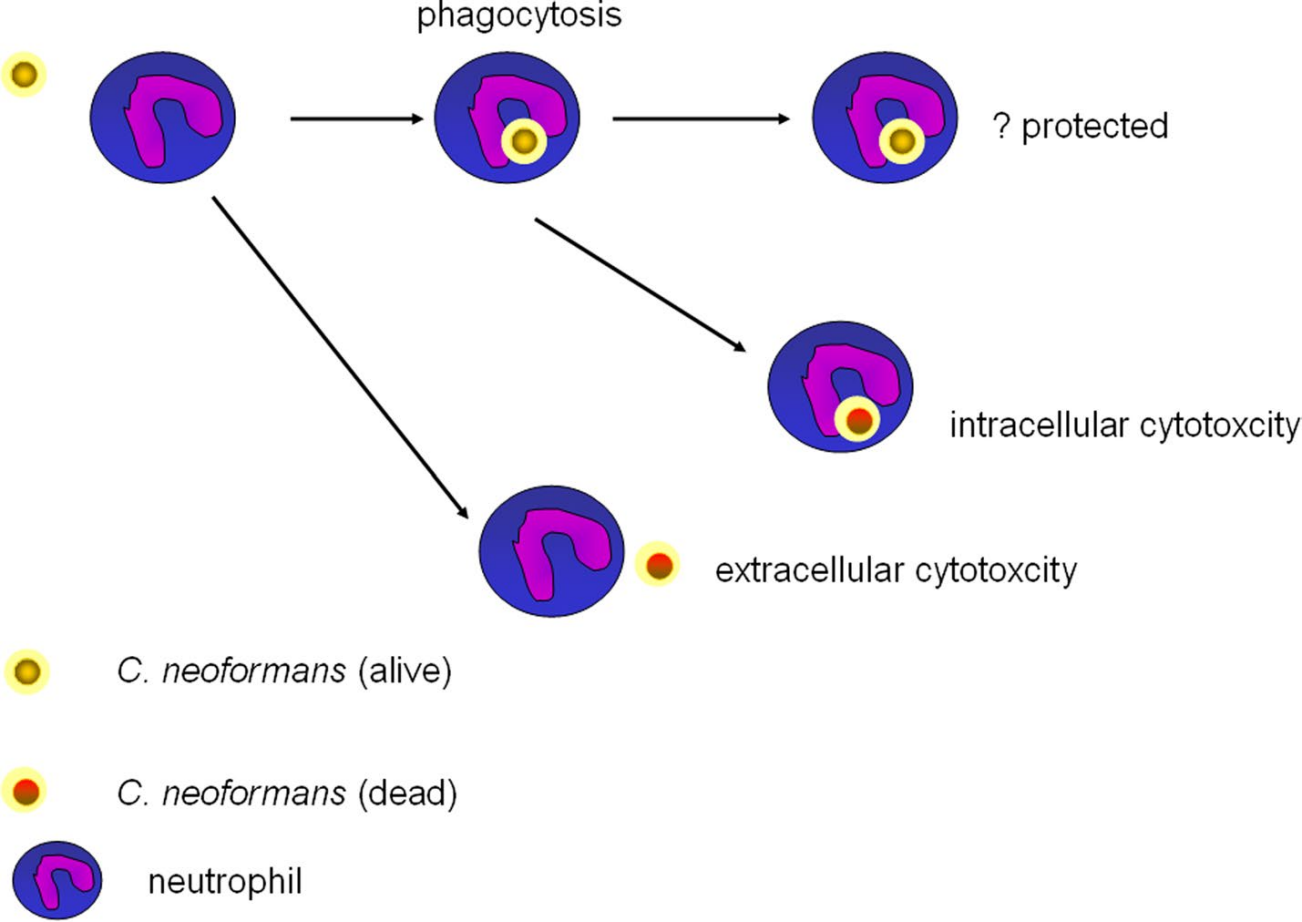
Roles of neutrophils in the Cn pathogenesis. Neutrophil could kill Cn extracellularly or intracellularly. Meanwhile, limited evidences argue that neutrophil may also protect the internalized Cn.

Neutrophils are not only phagocytes but also the modulators of immune responses. Depletion of neutrophils results in a Th2 response and renders mice susceptible to *Candida albicans* infection (Romani et al. 1997). However, roles of neutrophil depletion on the infection of Cn are more complicated. Mice infected with Cn intratracheally survive significantly longer if neutrophils are transiently depleted 24 h before the fungus inoculation, which is associated with the higher levels of IL-10, TNF-α, IL-4 and IL-12 in the lung (Mednick et al. 2003). In contrast to the protective role of neutrophil depletion, mice defective in neutrophil-specific enzyme MPO are hyper-susceptible to Cn, which might result from higher level of IL-4 and reduced production of IL-12, IFN-γ in the lung (Aratani et al. 2006). To add complexity more, neutrophil depletion in the mice infected with Cn expressing IFN-γ results in increased IL-17A production from γδT cells, but has no role on the fungus burden (Wozniak et al. 2012) (Fig. 2).

## Dendritic cells

Upon Cn airway infection, CCR2 mediates the recruitment of Ly6G^high^ monocytes (Osterholzer et al. 2009a), which differentiate into dendritic cells (DCs) and contribute to the Th1 response (Osterholzer et al. 2008). As the most potent antigen presenting cells, DCs internalize Cn via mannose receptor and FcγR-II in vitro (Syme et al. 2002) and in vivo (Wozniak et al. 2006), which is partially inhibited by the capsule (Vecchiarelli et al. 2003). In contrast, mannoproteins, interacting with CD206 and CD209 (Mansour et al. 2006), promote the maturation of dendritic cells (Pietrella et al. 2005). In the CD206 deficient mice, maturation of dendritic cells upon mannoproteins, however, is not hampered (Dan et al. 2008). Complements and specific antibodies promote the phagocytosis of Cn by dendritic cells (Kelly et al. 2005). Following phagocytosis, DCs kill the intracellular Cn via the fusion of endosome and lysosome and present antigens to T cells (Wozniak and Levitz 2008). The direct cytotoxicity of DCs against Cn is further confirmed in a recent study showing that purified lysosomal enzymes, specifically cathepsin B, inhibit cryptococcal growth in vitro (Hole et al. 2012).

**Fig. 3:**
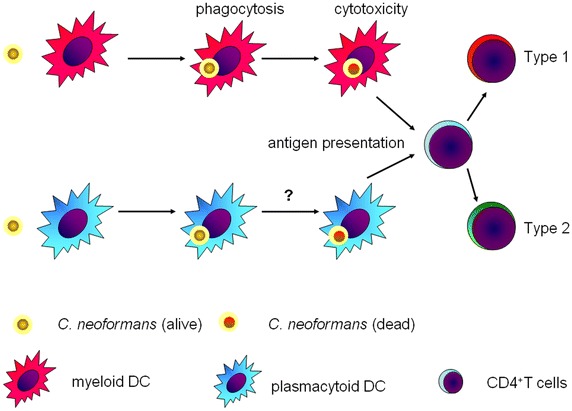
Roles of dendritic cells in the Cn pathogenesis. As most powerful antigen presenting cells, myeloid dendritic cells process and present Cn antigen to CD4^+^ T cells for the differentiation of cytotoxic Th1 cells. In contrast, plasmacytoid dendritic cells induce the non-protective Th2 cells. No evidence for the survival or death of Cn inside the plasmacytoid dendritic cells has been yet provided in the literature.

In the lymphnodes, Langerhans cells and myeloid DC induce protective CD4^+^ T cell responses against Cn (Bauman et al. 2000), which is augmented by TNF-α (Bauman et al. 2003). Accordingly, TNF-α deficiency decreases mature dendritic cell trafficking and produces a chronic Cn infection (Herring et al. 2005). Compared with myeloid DCs, plasmacytoid DCs induce non-protective immune response against Cn (Bauman et al. 2000; Siegemund and Alber 2008). Besides, non-protective Th2 responses could also be induced by immature dendritic cells in the lung, which are promoted by Cn urease (Osterholzer et al. 2009b) (Fig. 3).

## Endothelial cells

**Fig. 4:**
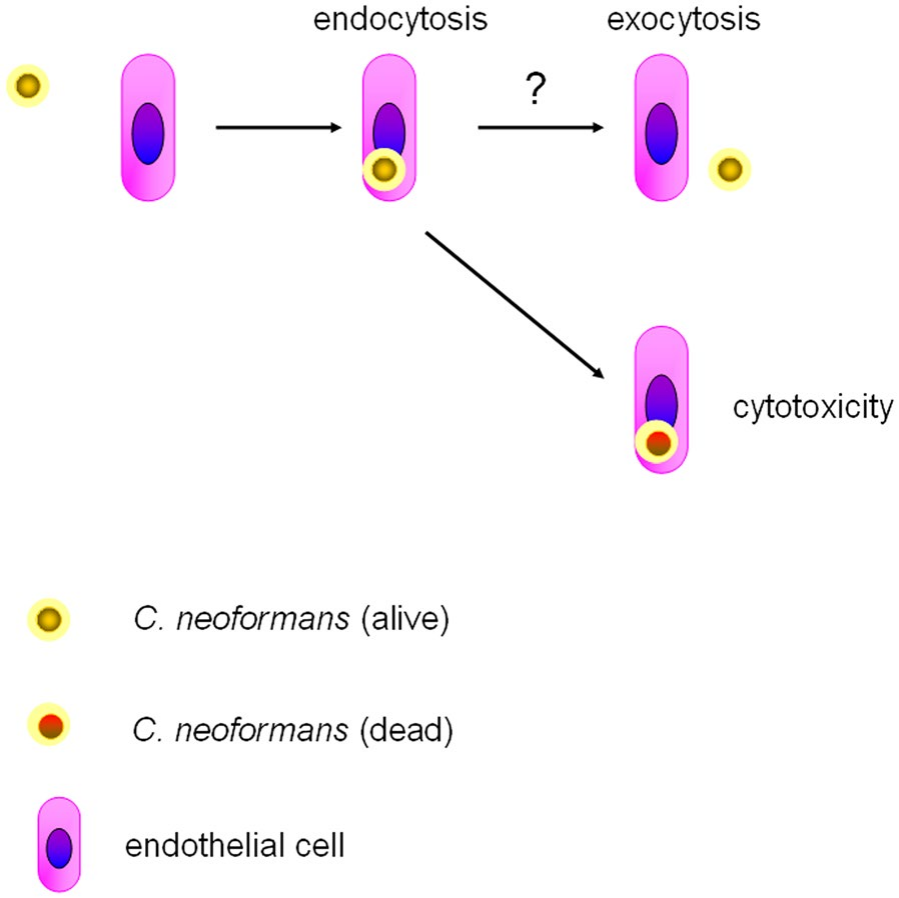
Roles of endothelial cells in the Cn pathogenesis. The major route for the BBB crossing by free Cn is transcellular pathway. As ferrymen, human brain microvasculature endothelial cells also kill the intracellular Cn via unidentified mechanisms.

Different from monocytes/macrophages, neutrophils and dendritic cells, endothelial cells are not professional phagocytes. Yet, Cn is observed in the brain endothelial cells of infected mice (Chretien et al. 2002). In vitro, free Cn could be surrounded by microvillus-like membrane protrusions and subsequently internalized by brain endothelial cells (Chang et al. 2004). Multiple molecules are engaged in the interactions between endothelial cells and extracellular Cn. Hyaluronic acid (HA) from Cn is the ligand of CD44 on the endothelial cells (Jong et al. 2008). In the process of transcellular migration, CD44 is co-localized with phosphorylated caveolin-1, forming thread-like structure (Long et al. 2012) and promoting the lipid raft-dependent endocytosis (Huang et al. 2011). Fungal burden in the brain is significantly decreased in the CD44 deficient mice intravascularly infected with Cn (Jong et al. 2012). Besides HA-CD44 pathway, urease (Shi et al. 2010), plasmin (Stie and Fox 2012), or metalloprotease Mpr1 (Vu et al. 2014) promotes migration of Cn across the brain endothelium by facilitating attachment of cryptococci to the endothelial cells, which induces the cytoskeleton remodeling and internalization (Vu et al. 2013). Of note, some other fungi, for example, *Candida albicans* also invades brain endothelial cells via endocytosis (Filler and Sheppard 2006). Thus, it would be interesting to explore whether or not brain endothelial cells express some unique receptors for Cn. Although there is no evidence, it is hypothesized that the internalized Cn would be expulsed from endothelial cells into the brain neuropil. Mechanisms for the Cn exocytosis from the endothelial cells are largely unknown. Moreover, human brain endothelial cells (Filler and Sheppard 2006) but not human umbilical vein endothelial cells (Roseff and Levitz 1993) or mouse brain endothelial cells (Sabiiti and May 2012) may also have the capability to kill the internalized Cn (Fig. 4).

## Conclusions

Serological evidences suggest that people may have been infected with environmental Cn in early childhood (Goldman et al. 2001). Most of Cn infection might be asymptomatic unless the immune defense is significantly suppressed (e.g., organ transplant patients) or defective (e.g., AIDS patients). Cn is overwhelmingly distributed in the environment and may hide in the people with weakened immune system (Saha et al. 2007). However, only a fraction of organ transplant patients or AIDS patients would develop fatal cryptococcosis. Are there any unidentified factors breaking the fine balance between Cn and phagocytes in the lung or even in the brain? How do phagocytes dynamically interact with Cn in the brain vasculature? How do phagocytes containing Cn transmigrate to the brain parenchyma? How does the free Cn escape from the BBB endothelial cells? Obviously, roles of phagocytes in cryptococcosis deserve further investigation.

## Abbreviations

Cn: *Cryptococcus neoformans*
BBB: blood–brain-barrier
CNS: central nervous system
App1: antiphagocytic protein 1
PLB1: phospholipase B1
G-CSF: granulocyte colony-stimulating factor
GXM: glucuronoxylomannan
C3: complement 3
MPO: myeloperoxidase
SMS: sphingomyelin synthase
DCs: dendritic cells
HA: hyaluronic acid
